# Modified mRNA Formulation and Stability for Cardiac and Skeletal Muscle Delivery

**DOI:** 10.3390/pharmaceutics15092176

**Published:** 2023-08-22

**Authors:** Magdalena M. Żak, Keerat Kaur, Jimeen Yoo, Ann Anu Kurian, Matthew Adjmi, Gayatri Mainkar, Seonghun Yoon, Lior Zangi

**Affiliations:** 1 Cardiovascular Research Institute, Icahn School of Medicine at Mount Sinai, New York, NY 10029, USA; 2 Department of Genetics and Genomic Sciences, Icahn School of Medicine at Mount Sinai, New York, NY 10029, USA; 3 Black Family Stem Cell Institute, Icahn School of Medicine at Mount Sinai, New York, NY 10029, USA

**Keywords:** mRNA stability, mRNA delivery, lipid nanoparticle-encapsulated mRNA

## Abstract

Directly injecting naked or lipid nanoparticle (LNP)-encapsulated modified mRNA (modRNA) allows rapid and efficient protein expression. This non-viral technology has been used successfully in modRNA vaccines against SARS-CoV-2. The main challenges in using modRNA vaccines were the initial requirement for an ultra-cold storage to preserve their integrity and concerns regarding unwanted side effects from this new technology. Here, we showed that naked modRNA maintains its integrity when stored up to 7 days at 4 °C, and LNP-encapsulated modRNA for up to 7 days at room temperature. Naked modRNA is predominantly expressed at the site of injection when delivered into cardiac or skeletal muscle. In comparison, LNP-encapsulated modRNA granted superior protein expression but also additional protein expression beyond the cardiac or skeletal muscle injection site. To overcome this challenge, we developed a skeletal-muscle-specific modRNA translation system (skeletal muscle SMRTs) for LNP-encapsulated modRNA. This system allows controlled protein translation predominantly at the site of injection to prevent potentially detrimental leakage and expression in major organs. Our study revealed the potential of the SMRTs platform for controlled expression of mRNA payload delivered intramuscularly. To conclude, our SMRTs platform for LNP-encapsulated modRNA can provide safe, stable, efficient and targeted gene expression at the site of injection.

## 1. Introduction

Nucleoside-modified mRNA (modRNA) was used globally for vaccination against COVID-19 and has been shown to be a safe, highly efficient, and clinically relevant approach for gene delivery [1,2]. Among other applications, our previous work demonstrated that modRNA has therapeutic potential for ischemic heart diseases. Naked modRNA (suspended in sucrose citrate buffer) delivery in vivo immediately induces high protein expression in several cardiac cell types, including cardiomyocytes (CMs) [3,4,5,6,7,8]. Several modifications to mRNA have been applied to decrease immunogenicity and improve translation in cardiac cells. We have shown that in cardiac tissue, replacing 100% of Uridine-5′-Triphosphate (uridine) with N1-Methylpseudouridine-5′-Triphosphate (1-mψU, pseudouridine) in modRNA synthesis renders it non-immunogenic. Additionally, pseudouridine-based modRNA shows improved RNAase resilience, allowing for higher protein translation when compared to uridine-based mRNA [6,9]. To date, modRNA technology has been used in mouse models of ischemic heart disease to promote cardioprotection [10], CM proliferation [11,12], cardiovascular restoration [4,13], and attenuation of cardiac fibrosis and hypertrophy post-ischemic injury [14].

While the two lipid nanoparticle (LNP)-encapsulated modRNA COVID-19 vaccines allowed rapid vaccination around the world, they initially required extremely cold (−80 °C) storage conditions [15], which limited their distribution and use in low-resource countries with limited cold chain storage. Since the first launch of modRNA vaccines, more research was performed regarding storage conditions with the current recommendation for LNP-encapsulated BioNTech/Pfizer’s COVID-19 vaccine being up to 30 days at 4 °C for sealed vials [16]. Despite this limitation, LNP-encapsulation of modRNA allows for more efficient protein expression in vivo when compared to naked modRNA making it an important delivery vehicle for the vaccine. However, the pharmacokinetic distribution of modRNA-LNP formulation post intracardiac or intramuscular injection still needs to be elucidated to understand potential secondary effects. For instance, vaccine-associated myocarditis is a pathological condition observed in COVID-19 modRNA vaccines at a rate of 0.0002% [17,18]. In comparison, vaccine-associated myocarditis for the smallpox vaccine has a frequency of 0.5–0.01% [19,20] and only anecdotal case reports for other vaccines [21]. The clinical manifestations of myocarditis are reported to arise 3–5 days after the second modRNA vaccine dose or occasionally after the first dose. One explanation for myocarditis after delivery of LNP-encapsulated modRNA vaccine is the migration of LNPs into the vascular system, eventually reaching the heart [18]. Therefore, potential LNP migration poses a challenge that should be understood and considered for future modRNA formulations and delivery systems.

To better understand the fate of naked versus LNP-encapsulated RNA, as well as properties of synthetic modRNA (containing pseudouridine) versus synthetic non-modified mRNA (containing uridine), we aimed to explore the difference in thermal stability and expression biodistribution patterns among these. Such baseline research is still needed during early stages of development for modRNA technology, and may contribute to advancing the field of modRNA vaccines and therapeutics [15].

We recently demonstrated that our cardiomyocyte-specific modRNA translation system for intracardiac delivery (CM SMRTs) of naked modRNA allows for selective gene expression of naked modRNA in cardiomyocytes [12,22]. The CM SMRTs modRNA constructs enable cell-specific mRNA expression by taking advantage of cellular microRNA (miR) profiles. Our CM-specific modRNA constructs carry both CM specific miR-208a and miR-1 recognition elements, which we found to enhance expression of target genes in CMs. Based on this and our previous results, we designed a skeletal-muscle-specific SMRTs using miR-1, which is highly expressed in skeletal muscle [23]. The SMRTs system contains two modRNA molecules that work as an on/off system. One modRNA molecule carries a miR-1 recognition element and the L7Ae gene, which codes for a protein that selectively binds to a K-turn motif. The second modRNA molecule carries a K-turn sequence and the sequence for the gene of interest. In the presence of miR-1, L7Ae expression is suppressed, which allows for transgene expression. Conversely, in the absence of miR-1, L7Ae gene is translated into a protein that binds to the K-turn motif, which then blocks the expression of the transgene in cells other than the skeletal muscle.

We hypothesized that the developing organ and cell-specific modRNA formulations that can be easily stored in common temperature conditions for long periods of time would improve the versatility of modRNA vaccines and therapeutics. In pursuit of this, we conducted this study to evaluate the thermal stability of naked and LNP-encapsulated modRNA or SMRTs in different storage conditions and their biodistribution in the body post cardiac and skeletal intramuscular injections.

## 2. Materials and Methods

### 2.1. Construction of Templates and Synthesis of Synthetic of mRNA and Synthetic modRNA

Clean PCR products generated with plasmid templates, purchased from GenScript, were used as the template for mRNA. modRNAs were generated by transcription in vitro with a customized ribonucleoside blend of ARCA; 3′-O-Me-m^7^G(5′)ppp(5′)G (Trilink Biotechnologies, San Diego, CA, USA); Guanosine-5′-triphosphate (GTP); Adenosine triphosphate (ATP); Cytidine triphosphate (CTP); Uridine-5′-triphosphate (UTP) (in case of synthetic mRNA) (Life Technologies, Carlsbad, CA, USA); and N1-Methylpseudouridine-5′-triphosphate (Trilink Biotechnologies) in the case of synthetic modRNA [24]. The mRNA was purified either with the MEGA clear kit (Life Technologies) according to the manufacturer’s instructions or using Amicon Ultra-4 Centrifugal Filter Unit 4 mL,10 kDa (Millipore Sigma, Burlington, MA, USA) and treated with Antarctic Phosphatase (NEB). Next, the mRNA was re-purified with the MEGA clear kit. The mRNA was quantified using a Nano Drop spectrometer (Thermo Scientific, Waltham, MA, USA), precipitated with ethanol and ammonium acetate, and re-suspended in 10 mM Tris-HCl and 1 mM EDTA. The open reading frame for the Luc and nGFP modRNA is listed below. Endogenous Luc mRNA is a mixture of mRNA enriched with luciferase gene, obtained by isolation of RNA form 4T1-Luc cell line (ATCC #CRL2539LUC2).

### 2.2. BMDM Differentiation and FACS Staining

Femurs and tibias from 8–10-week-old BL6 mice were flushed with RPMI medium (Gibco #72400-047, Billings, MT, USA) and the resulting single-cell suspension was filtered using a 70 µm cell strainer (Corning #431751, Corning, NY, USA) and centrifuged for 5 min at 420× *g*. Red blood cell lysis was performed by pouring 4 mL of sterile H_2_O followed immediately by 46 mL of sterile PBS and centrifuged for 5 min at 420× *g*. BMDM were cultured in Macrophage Serum Free Media (Gibco #12065-074) supplemented with 100 ng/mL of murine M-CSF (Peprotech #315-02, Rocky Hill, NJ, USA) and pen–strep. After overnight incubation, floating cells were harvested and plated on two new cell culture plates and 30% of fresh medium was replaced every other day. BMDM were harvested on day 8. To confirm differentiation of bone marrow cells into BMDM, FACS staining was performed on day 8. Cells and controls were stained with CD11b-APC (BioLegend, San Diego, CA, USA, #101211) and F4/80-PE-Cy7 (BioLegend, #123113). For single-color controls, Anti-Rat Ig, κ/Negative Control Compensation Particles Set was used (BD Biosciences, 552844). Data were acquired using Aurora (Cytek, Fremont, CA, USA) Spectral Flow Cytometer. Macrophages were defined as CD11b^+^ F4/80^+^.

### 2.3. Cell Transfections and Bioluminescent Imaging

HEK293T cells were cultured using DMEM media supplemented with 10% FBS and pen–strep. BMDM were cultured as described above. A total of 50,000 cells per well were plated on a black 24-well plate (Perkin Elmer, Waltham, MA, USA) one day before transfections. Synthetic Luc modRNA, synthetic Luc mRNA or endogenous Luc mRNA samples stored under various conditions were used to transfect the cells. A total of 0.5 µg of synthetic Luc modRNA, synthetic Luc mRNA, or 5 µg of endogenous Luc mRNA was used per well, with jetMESSENGER transfection reagent (Polyplus, #101000005), following the manufacturer’s protocol. At 6 h post transfection, medium was changed, and bioluminescent imaging was performed 24 h later. Cell culture medium containing 150 µg/mL of D-Luciferin Potassium Salt (Perkin Elmer, #122799) was added, and cells were imaged every 1 min using IVIS Spectrum In Vivo Imaging System (Perkin Elmer) until maximum luminescence was reached. To assess BMDM cell survival post transfection, BMDM cells were transfected with synthetic Luc modRNA, synthetic Luc mRNA, or endogenous Luc mRNA as described above. At 24 h after transfections, cells were fixed with 4% PFA and stained with DAPI. Cell survival was calculated as a percentage of DAPI^+^ cells to non-transfected cells.

### 2.4. In Vivo modRNA Injections

In vivo gene delivery was performed according to previously published methods [25]. modRNA delivered naked was diluted in a sucrose citrate buffer containing 20 µL of sucrose in nuclease-free water (0.3 g/mL), with 20 µL of citrate (0.1 M [pH 7]; Sigma, Kawasaki, Japan) mixed with 30 µg modRNA in saline to a total volume of 60 µL. For liposome-based delivery, modRNA was encapsulated with JetRNA (Polyplus, Illkirch, France) reagent according to the manufacturer’s recommendation and was concentrated using concentration filters (Millipore, Burlington, MA, USA) to the approximate volume of 60 µL. LNPs were formulated via microfluidic mixing of one part ethanol phase containing the lipids and three parts aqueous phase containing the mRNA or SMRTs. In the case of SMRTs, the solution of two modRNA (L7Ae and Luciferase) was mixed and formulated to LNPs together. The ethanol phase contains the ionizable lipid ALC0315, cholesterol, DSPC and ALC0159 at a molar ratio of 46.3:42.7:9.4:1.6. The aqueous phase consists of the Luc modRNA in 25 mM Citrate buffer pH 4. Following microfluidic mixing, the nanoparticle solution was concentrated to 60 µL volume using Amicon Ultra filters (Millipore, Burlington, MA, USA). After the transfection mix was concentrated, all three formulations were directly injected into heart muscle (two in the left ventricle and one in the apex), 20 µL at each site. For skeletal-muscle injections, 60 µL of various modRNA formulations were injected into the femur skeletal muscle.

### 2.5. Quality Control of LNP-RNA Formulations

After formulation, LNPs were separated by following storage conditions: time 0 (immediately after formulation), 1 h at RT, 1 day at RT, 7 days at RT, 7 days 4 °C, 7 days at −80 °C, 7 days at −20 °C and 14 days RT. For each set of conditions, LNP samples were tested for size, polydispersity index (PDI) and encapsulation efficiency (EE). Hydrodynamic diameter and PDI were measured using Dynamic Light Scattering (DLS) particle size analyzer (Anton Paar Particle Analyzer Litesizer 500) at a 90° scattering angle. RNA encapsulation efficiency was measured using the Invitrogen Quant-iT RiboGreen RNA Assay Kit (#R11490) according to manufacturer’s protocol.

### 2.6. Bioluminescence Imaging

Luc modRNA, stored under different conditions and encapsulated with diverse delivery reagents, was directly injected into the myocardium or the quadriceps femoris muscle of CFW mice. Bioluminescence imaging of the transfected cells or injected mice was taken 24 h post injections. Prior to bioluminescence imaging, mice were anesthetized with isoflurane (Abbott Laboratories), and D-Luciferin Potassium Salt (Perkin Elmer, #122799) was injected intraperitoneally at 150 mg/kg body weight. Mice were imaged using an IVIS100 charge-coupled device imaging system every 2 min until the Luc signal reached a plateau. Imaging data were analyzed and quantified with Living Image software 2.0. Muscle tissues from mice injected with only Luciferin served as a baseline reading for Luc expression.

## 3. Results

### 3.1. Synthetic modRNA but Not Synthetic Non-Modified mRNA or Endogenous mRNA Translates Well in Primary Cell Culture

Synthetic mRNA (produced using the in vitro transcription (IVT) method), containing uridine and modRNA containing modified uridine (pseudouridine) has been used with transfection reagents for gene delivery to cell lines and primary cell cultures for many years [9,26]. Such mRNAs are typically stored at −80 °C [27,28,29] and thawed before being mixed with the transfection reagent. Our data (Figure S1) and previous reports [6,9] show that synthetic modRNA, unlike synthetic non-modified mRNA or endogenous mRNA, translates well in both cell line (HEK293) and a primary cell culture (bone marrow-derived macrophages (BMDM)). We demonstrated that transfecting cells using synthetic modRNA and non-modified mRNA, produce similar protein levels in the HEK293T cell line but non-modified mRNA gave significantly less translation in BMDM (Figure S1C,D). Additionally, we showed that transfection with synthetic non-modified mRNA or endogenous mRNA caused significantly more toxicity in BMDM cells when compared to transfection with synthetic modRNA (Figure S1E).

### 3.2. Naked modRNA Expresses Predominantly in the Injection Area and Keeps Its Integrity When Stored for One Day at Room Temperature (RT) or up to Seven Days at 4 °C

**Figure 1:**
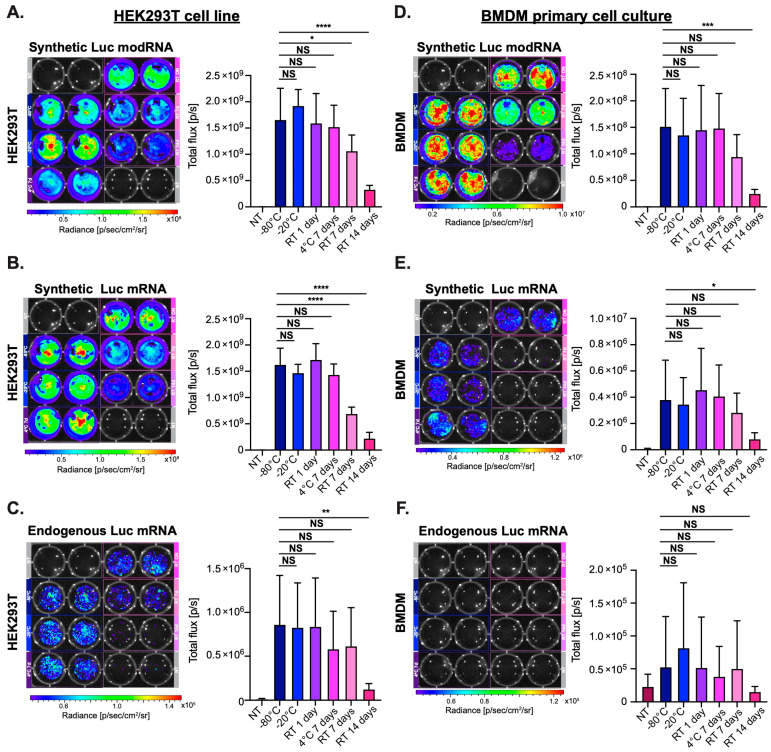
In vitro translation efficiency of synthetic Luc modRNA, synthetic Luc mRNA and endogenous Luc mRNA in HEK293T cell line or primary culture (BMDM) after storage in various temperature conditions. (**A**–**C**). HEK293T cell line was transfected with synthetic Luc modRNA (**A**), synthetic Luc mRNA (**B**) or endogenous Luc mRNA (**C**) stored in various conditions. Bioluminescence signal was measured 24 h post transfection. (**D**–**F**). BMDM primary cell culture was transfected with synthetic Luc modRNA (**D**), synthetic Luc mRNA (**E**) or endogenous Luc mRNA (**F**) stored at different conditions. Bioluminescence signal was measured 24 h post transfection. One-way ANOVA and Tukey’s Multiple Comparison Test, n = 8, in three independent experiments. NS: *p* > 0.05; * *p* ≤ 0.05; ** *p* ≤ 0.01; *** *p* ≤ 0.001, **** *p* ≤ 0.0001.

It has previously been suggested that modRNA must be kept at −80 °C to prevent its degradation. We wanted to evaluate how storing mRNA in differing conditions affects its stability and potency. To determine this, we measured the integrity and translation ability of synthetic luciferase (Luc) modRNA, synthetic Luc mRNA or endogenous Luc mRNA stored under different conditions, including RT (~24 °C) for one, seven, or fourteen days, as well as seven days at 4 °C or −20 °C. Quality control of different Luc mRNA preparations in various storage conditions showed that all types of Luc mRNA have similarly high integrity when kept at −20 °C or 4 °C, up to seven days, or one day at RT. Conversely, synthetic non-modified Luc mRNA integrity was compromised after storage at RT for seven days with even further degradation at 14 days (Figure S2). Next, we evaluated the translation ability of different Luc mRNA kept under different storage conditions in a cell line (HEK293T) or in primary cell culture (BMDM). We transfected the cells with different mRNA using a commercially available mRNA transfection reagent (jetMESSENGER), and determined their translation by measuring the bioluminescence (BL) signal (Figure 1). Our results revealed that endogenous mRNA has poor translation capacity in a HEK293T cell line and almost no ability to translate in a BMDM primary cell culture (Figure 1C,F). Both synthetic Luc modRNA and non-modified mRNA translated similarly in the HEK293T cell line and had a significantly reduced BL signal when either modRNA or mRNA was kept at RT for 7 or 14 days (Figure 1A,B). In a BMDM primary cell culture, Luc modRNA allows for an over 300 times higher protein expression than Luc non-modified mRNA (Figure 1D,E). As in the HEK293T cell line, both modRNA and non-modified mRNA show significantly reduced translation when kept at RT for 14 days (Figure 1D,E).

**Figure 2:**
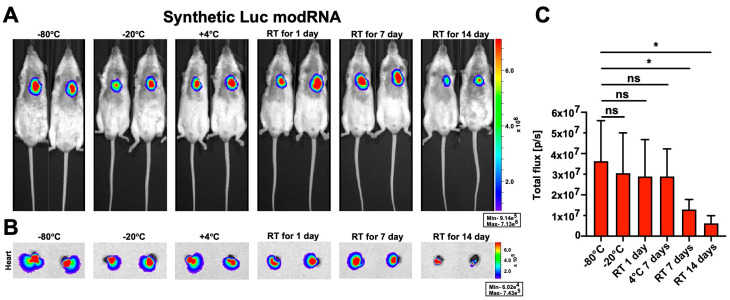
Stability and biodistribution of naked modRNA when delivered intracardially. (**A**). Delivery of naked Luc modRNA shows translation only in the heart. Representative bioluminescence images of whole mice (**A**) and hearts (**B**) 24 h post delivery of synthetic Luc modRNA stored in different conditions. (**C**). Quantification of Luc expression in hearts isolated from mice injected with naked modRNA stored in different conditions. One-way ANOVA and Tukey’s Multiple Comparison Test, n = 5, in two independent experiments. ns: *p* > 0.05; * *p* ≤ 0.05.

Next, we evaluated stability and biodistribution of naked modRNA in vivo. We have previously shown the advantages of delivery of naked modRNA into cardiac tissue [6]. There is currently a clinical trial investigating the beneficial effect of VEGF-A modRNA delivered naked to the heart in patients with ischemic heart disease [30]. To further define the dynamics of naked modRNA, samples were stored under different storage conditions and translation was evaluated post-injection (Figure 2). We showed that direct intramyocardial injection of naked modRNA leads to translation predominantly in the heart 24 h post injection (Figure 2A,B). Similarly with our in vitro results in cell cultures, modRNA kept at RT for 7 or 14 days had significantly less translation at the site of injection (i.e., the heart, Figure 2C).

### 3.3. Direct Myocardial Injection of ALC-0315-Encapsulated modRNA Causes Protein Translation at the Injection Site and in Major Mouse Organs

**Figure 3:**
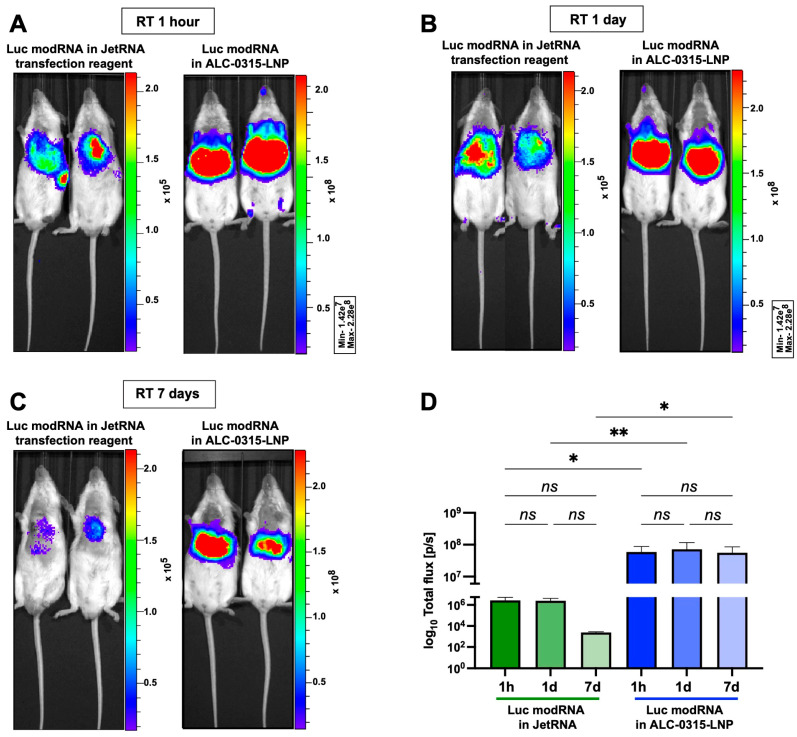
LNP encapsulation of modRNA grants superior protein expression in vivo. Comparison of translation efficiency of Luc modRNA encapsulated using JetRNA, a liposome-based transfection reagent for in vivo delivery, or lipid nanoparticles (ALC-0315) at different time points post formulation. Bioluminescent imaging was performed 24 h after intracardiac injection. Representative images of mice injected one hour (**A**), one day (**B**) or seven days (**C**) after modRNA encapsulation. (**D**) Quantification of the bioluminescence signal comparing different mRNA vehicles and time points. One-way ANOVA and Tukey’s Multiple Comparison test. ns: *p* > 0.05; * *p* ≤ 0.05; ** *p* ≤ 0.01.

We have shown that intracardiac injection of naked modRNA allows translation predominantly at the site of injection (Figure 2), also shown in previous publications [4,6,12,22]. Here (Figure 3), we wanted to evaluate the difference in translation, stability and biodistribution of modRNA when delivered in vivo using a commercially available non-LNP transfection reagent (JetRNA) or when encapsulated in LNP. For LNP preparation, we used ionizable lipid ALC-0315 (ALC-0315-LNP), used in the BNT162b2 vaccine (Pfizer/BioNTech) [31]. Quality control of freshly prepared LNP-modRNA formulations showed a size of 67.9 ± 3.0 nm when using Luc modRNA and 63.1 ± 1.0 nm for nGFP modRNA, suggesting that modRNA length (1653nt for Luc mRNA and 801nt for nGFP mRNA) plays a minimal a role in the overall size of LNP-RNA formulation. Similarly, RNA encapsulation efficiency (EE) ranges from 93.2 ± 1.6% for Luc and 93.2 ± 0.1% for nGFP and polydispersity index (PDI) is 21.0 ± 2.0% for Luc and 20.6 ± 4.8% for nGFP (Figure S3). Additionally, we measured changes in properties of LNP-modRNA formulations after storage in various conditions. We observed no changes in size, EE and PDI when stored for up to 14 days at RT. However, freezing of LNP-modRNA formulations either at −20 °C or −80 °C for 7 days compromised their integrity (Figure S3). Typically, sucrose is used for cryopreservation of LNP-mRNA formulations [32]. Next, we compared the vehicles for in vivo mRNA delivery. We observed that modRNA formulations using JetRNA show lower translation efficiency post intracardiac injection when compared to ALC-0315-LNP modRNA (Figure 3D). Importantly, when using either vehicle, we observed protein expression in the injection site (heart) as well as in other major mouse organs (especially the liver, Figure 3A–C). Our results also indicate that vehicle-Luc mRNA formulations preserve modRNA translation capacity when stored at RT for up to 7 days before delivery to the heart (Figure 3D). This observation is consistent with the results of quality control of LNP-RNA formulations (Figure S3).

### 3.4. Direct Intramuscular Injection of LNP-Encapsulated Skeletal Muscle SMRTs Allows Efficient Translation Primarily at the Site of Injection, and Not in Other Mouse Organs

**Figure 4:**
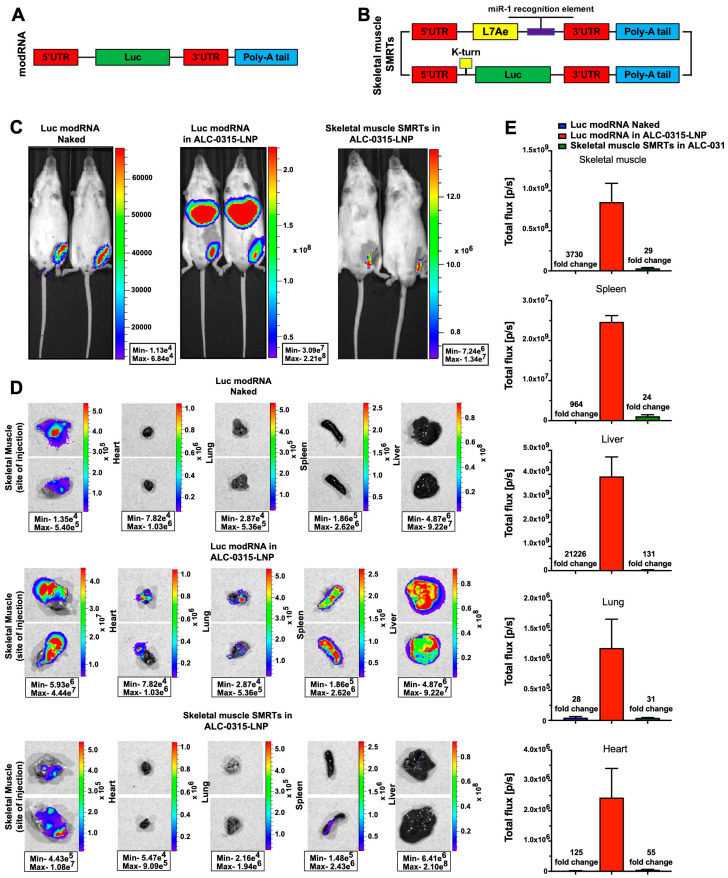
LNP-encapsulated skeletal muscle-specific SMRTs allows injection site-restricted expression of modRNA post intramuscular injection. Scheme of modRNA (**A**) and skeletal muscle SMRTs (**B**) constructs. Expression pattern of modRNA Luc in sucrose citrate buffer (naked) or LNP-formulated modRNA Luc or skeletal muscle SMRTs Luc was evaluated 24 h post direct injection into skeletal muscle. Representative bioluminescence images of mice (**C**) and main organs (**D**) post intramuscular injection. (**E**) Comparison of Luc expression in femoral muscle, heart, lung, spleen, and liver between different formulations and RNA constructs.

modRNA has high translation ability in both skeletal and cardiac muscle [4,13]. As ALC-0315-LNP was used in the SARS-CoV-2 vaccines to deliver spike modRNA into skeletal muscle [31], and because our data (Figure 3) suggest that modRNA encapsulated in ALC-0315-LNP translate beyond the injection site, we wanted to determine if modRNA encapsulated in ALC-0315-LNP translates outside the skeletal muscle injection site. We observed that when injected directly into the skeletal muscle, naked modRNA showed a 3730-fold lower translation when compared to ALC-0215-LNP modRNA (Figure 4C). However, while naked modRNA delivery allowed translation predominantly at the injected site, modRNA encapsulated in ALC-0315-LNP produced a high expression in distant mouse organs, especially the liver and spleen (Figure 4D).

Skeletal muscles express miR-1 [23]. We, therefore, created a skeletal muscle SMRTs based on miR-1 to translate Luc preferably in skeletal muscle but not in other non-muscular organs (Figure 4A,B). We showed that skeletal muscle SMRTs encapsulated in ALC-0315-LNP had significantly reduced protein expression in the liver, spleen and the heart, suggesting that the use of LNP-encapsulated skeletal-muscle SMRTs allows for efficient expression at the skeletal muscle injection site (~4 × 10^7^ p/s versus ~5.0 × 10^5^ for naked modRNA) with reduced expression in distant organs. These results indicate the importance of designing LNP-protected, cell-specific modRNA SMRT systems that allow for high translation efficiency limited to the injection site to avoid possible side effects.

## 4. Discussion

modRNA technology has been proven clinically relevant during the COVID-19 pandemic. Millions of modRNAs vaccines (made by Moderna and Pfizer/BioNTech) have been administrated successfully to avert SARS-CoV-2-related death and hospitalization. Due to this achievement, we are witnessing a paradigm shift in the way we employ genes that translate therapeutic proteins in the human body. We now rely less on viral vectors and more on non-viral gene delivery methods like modRNA.

We and others [9] have shown that modRNA, but not non-modified mRNA or endogenous mRNA, are suitable for efficient gene delivery in mammals. Non-modified mRNA causes increased toxicity in some cell types and significantly reduced protein translation (Figure 1 and Figure S1). Further, non-modified mRNA was shown to activate RIG-1, TLR7 and/or TLR8, which results in the production of type I interferons, reduced antigen translation and enhanced apoptosis pathways [9,33]. However, non-modified mRNA has been used in applications where the activation of the immune system is desirable, like in anticancer vaccines developed by BioNTech [34,35]. Our work has demonstrated that modRNA is a relatively stable molecule that can keep its integrity and translational ability when stored for up to seven days at −20 °C or at 4 °C or for one day at RT (Figure 1, Figure 2 and Figure S2). This input may improve the way we handle naked modRNA for research applications; shipping naked modRNA on ice (at 4 °C) is more straightforward and cost efficient than shipping on dry ice (at −80 °C).

Our data suggest that modRNA encapsulated in LNPs made with the same ionizable lipid (ALC-0315) used in the Pfizer/BioNTech vaccine preserves modRNA stability and transfection efficiency when stored up to seven days at RT (see Figure 3). Examination of bioluminescence signals in the heart post intracardiac injection revealed that LNP-encapsulated modRNA translation efficiency (BL 6 × 10^7^ p/s) is two-fold higher when compared to naked modRNA (BL 3 × 10^7^ p/s) and 20-fold higher when compared to the non-LNP vehicle, JetRNA (BL 3 × 10^6^ p/s). These observations suggested that LNP encapsulation was the most effective way to deliver modRNA in vivo, however, the potential problem with LNP-encapsulated modRNA is its biodistribution. We showed that modRNA in ALC-0315-LNP causes protein expression beyond the injection site; in both the heart and skeletal muscle (Figure 3 and Figure 4). This may lead to unwanted gene expression in distal organs and promote detrimental effects, especially with immunogenic payloads (as in vaccines). Accordingly, modRNA encapsulated in LNP should be designed to be expressed exclusively in specific organ/s and cell type/s to avoid unwanted side effects.

To achieve improved modRNA expression, we developed the SMRTs platform that enables modRNA translation into specific organs and cell types. This system, coupled with LNP technology, may pave the way for safer and more effective clinical use of modRNA. Skeletal muscle is the preferred organ for administration of vaccines, including modRNA vaccines [36], because it has an abundant blood supply, protective adipose tissue, fewer drainage channels, and a high regenerative capacity that allows it to retain injected materials for a longer period of time without substantial damage [37]. Other organs, like the heart, lack such regenerative capacity, therefore inflammation can have devastating effects, making those organs susceptible to unwanted side effects following vaccination. In our study, we injected 30 µg of modRNA (naked or encapsulated) per mouse into both cardiac and skeletal muscle; however, others [38,39] have used 1–15 µg per mouse. Recommended dosages of modRNA vaccines for adults are 30 µg of modRNA for the Pfizer/BioNTech’s and 100 µg for the Moderna’s [40,41]. This relatively high concentration of modRNA for a mouse model may contribute to the observed leakiness of the encapsulated modRNA to other organs. Our work shows (Figure 4) that direct injection of modRNA encapsulated in ALC-0315-LNP into skeletal muscle (a similar technique to the modRNA vaccination against SARS-CoV-2) generated modRNA translation in organs beyond the site of injection (e.g., liver). By contrast, injecting skeletal muscle SMRTs encapsulated in ALC-0315-LNP allowed translation primarily in skeletal muscle and not in other internal organs. However, this approach resulted in a 27-fold decrease in translation at the injection site compared to modRNA-LNPs, indicating further adjustments may be needed to improve the translation capacity of skeletal muscle SMRTs. Nevertheless, skeletal muscle SMRTs and the overall SMRTs platform enable safer and more precise delivery of modRNA to a specific tissue and organ. Looking forward, the next step will be to evaluate the SMRTs platform delivery system when injected systemically (e.g., intravenously): a modality that could target organs and cell types without directly injecting modRNA into target organs. Such systemic administration could replenish modRNA translation in organs and cell types and thus overcome the short pharmacokinetics of modRNA.

In summary, we believe that a better understanding of optimal storage conditions for LNP-modRNA formulations and focusing on designing modRNA constructs that allow organ- and cell-specific protein translation could unlock the staggering potential of modRNA therapeutics in the clinic.

## Data Availability

All modified mRNA (modRNA) vectors containing genes of interest noted in this paper will be made available to other investigators. My institution and I will adhere to the NIH Grants Policy on Sharing of Unique Research Resources including the “Sharing of Biomedical Research Resources: Principles and Guidelines for Recipients of NIH Grants and Contracts” issued in December 1999. Specifically, material transfers will be made with no more restrictive terms than in the Simple Letter Agreement or the UBMTA and without reach-through requirements. Should any intellectual property arise, which requires a patent, we would ensure that the technology remains widely available to the research community in accordance with the NIH Principles and Guidelines.

## Supplementary Materials

The following supporting information can be downloaded at: https://www.mdpi.com/article/10.3390/pharmaceutics15092176/s1, Figure S1: In vitro translation ability of synthetic Luc modRNA, synthetic Luc mRNA or endogenous Luc mRNA in HEK293T cell line or BMDM primary cell culture; Figure S2: Evaluation of the integrity of Luc RNA molecules; Figure S3: Evaluation of the integrity of synthetic modRNA-LNP formulations in various storage conditions; Table S1: Open reading frames.
