# Supplementary material for: Modified mRNA Formulation and Stability for Cardiac and Skeletal Muscle Delivery
Source: Pharmaceutics. 2023 Aug 22;15(9):2176. doi: 10.3390/pharmaceutics15092176 (PMC10535735; doi:10.3390/pharmaceutics15092176)
Supplement: Supplementary file 1 [file pharmaceutics-15-02176-s001.zip › pharmaceutics-2501336-supplementary.pdf]

Supplemental Material

1) Material and Methods

Table S1. Open reading frames.

Luc ORF	atggccgatgctaagaacattaagaagggccctgctcccttctaccctctggaggatggcaccgctggcgagcag<br>ctgcacaaggccatgaagaggtatgccctggtgcctggcaccattgccttcaccgatgcccacattgaggtggaca<br>tcacctatgccgagtacttcgagatgtctgtgcgcctggccgaggccatgaagaggtacggcctgaacaccaacc<br>accgcatcgtggtgtgctctgagaactctctgcagttcttcatgccagtgctgggcgccctgttcatcggagtggcc<br>gtggcccctgctaacgacatttacaacgagcgcgagctgctgaacagcatgggcatttctcagcctaccgtggtgtt<br>cgtgtctaagaagggcctgcagaagatcctgaacgtgcagaagaagctgcctatcatccagaagatcatcatcatg<br>gactctaagaccgactaccagggcttccagagcatgtacacattcgtgacatctcatctgcctcctggcttcaacga<br>gtacgacttcgtgccagagtctttcgacagggacaaaaccattgccctgatcatgaacagctctgggtctaccggcc<br>tgcctaagggcgtggccctgcctcatcgcaccgcctgtgtgcgcttctctcacgcccgcgaccctattttcggcaac<br>cagatcatccccgacaccgctattctgagcgtggtgccattccaccacggcttcggcatgttcaccaccctgggcta<br>cctgatttgcggctttcgggtggtgctgatgtaccgcttcgaggaggagctgttcctgcgcagcctgcaagactaca<br>aaattcagtctgccctgctggtgccaaccctgttcagcttcttcgctaagagcaccctgatcgacaagtacgacctgt<br>ctaacctgcacgagattgcctctggcggcgccccactgtctaaggaggtgggcgaagccgtggccaagcgctttc<br>atctgccaggcatccgccagggctacggcctgaccgagacaaccagcgccattctgattaccccagagggcgac<br>gacaagcctggcgccgtgggcaaggtggtgccattcttcgaggccaaggtggtggacctggacaccggcaaga<br>ccctgggagtgaaccagcgcggcgagctgtgtgtgcgcggccctatgattatgtccggctacgtgaataaccctg<br>aggccacaaacgccctgatcgacaaggacggctggctgcactctggcgacattgcctactgggacgaggacga<br>gcacttcttcatcgtggaccgcctgaagtctctgatcaagtacaagggctaccaggtggccccagccgagctgga<br>gtctatcctgctgcagcaccctaacattttcgacgccggagtggccggcctgcccgacgacgatgccggcgagct

	gcctgccgccgtcgtcgtgctggaacacggcaagaccatgaccgagaaggagatcgtggactatgtggccagcc<br>aggtgacaaccgccaagaagctgcgcggcggagtggtgttcgtggacgaggtgcccaagggcctgaccggca<br>agctggacgcccgcaagatccgcgagatcctgatcaaggctaagaaaggcggcaagatcgccgtgtaa
nGFP ORF	atggtgagcaagggcgaggagctgttcaccggggtggtgcccatcctggtcgagctggacggcgacgtaaacg<br>gccacaagttcagcgtgtccggcgagggcgagggcgatgccacctacggcaagctgaccctgaagttcatctgc<br>accaccggcaagctgcccgtgccctggcccaccctcgtgaccaccctgacctacggcgtgcagtgcttcagccg<br>ctaccccgaccacatgaagcagcacgacttcttcaagtccgccatgcccgaaggctacgtccaggagcgcaccat<br>cttcttcaaggacgacggcaactacaagacccgcgccgaggtgaagttcgagggcgacaccctggtgaaccgca<br>tcgagctgaagggcatcgacttcaaggaggacggcaacatcctggggcacaagctggagtacaactacaacagc<br>cacaacgtctatatcatggccgacaagcagaagaacggcatcaaggtgaacttcaagatccgccacaacatcgag<br>gacggcagcgtgcagctcgccgaccactaccagcagaacacccccatcggcgacggccccgtgctgctgcccg<br>acaaccactacctgagcacccagtccgccctgagcaaagaccccaacgagaagcgcgatcacatggtcctgctg<br>gagttcgtgaccgccgccgggatcactctcggcatggacgagctgtacaagggagatccaaaaaagaagagaa<br>aggtaggcgatccaaaaaagaagagaaaggtaggtgatccaaaaaagaagagaaaggtataa

Supplemental Figures

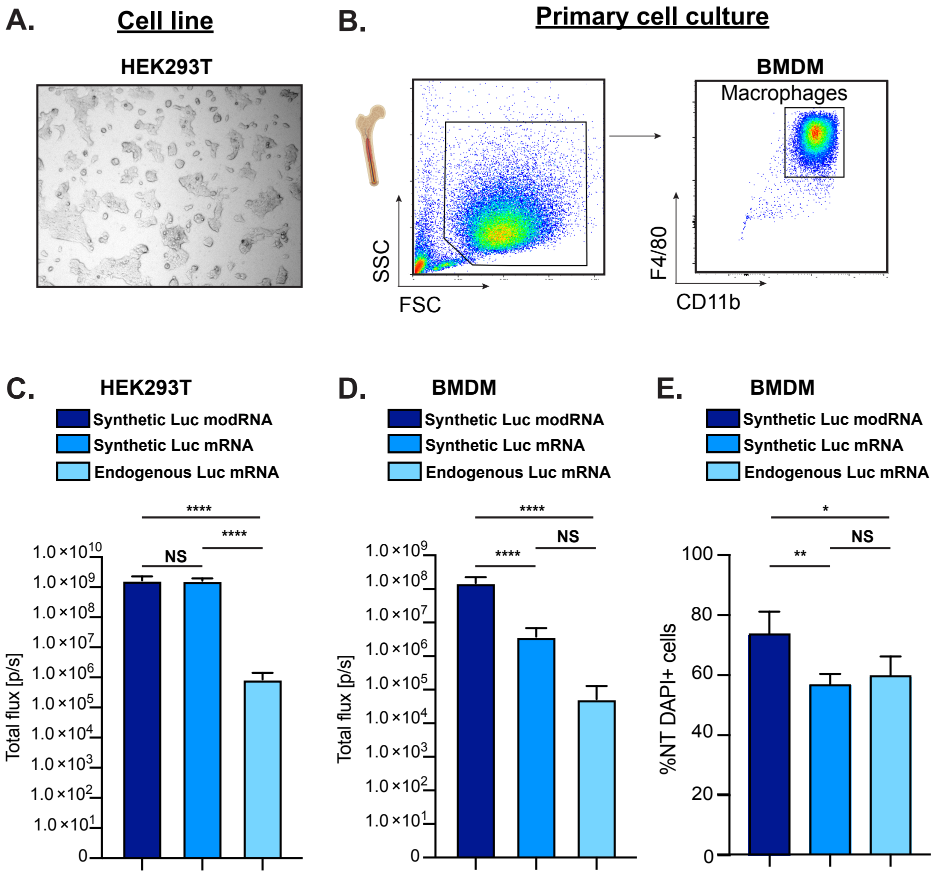

Figure S1: In vitro translation ability of synthetic Luc modRNA, synthetic Luc mRNA or endogenous Luc mRNA in HEK293T cell line or BMDM primary cell culture. A&B. HEK293T cell line and bone marrow-derived macrophage (BMDM) cell culture were used to assess the translation ability of synthetic Luc modRNA, synthetic Luc mRNA and endogenous Luc mRNA (isolated from 4T1-Luc cells) in the two different cell types. **C&D.** HEK293T cell line (**C**) and BMDM (**D**) were transfected with different mRNA Luc molecules. Bioluminescence signal was measured 24 h later. **E.** BMDM cell survival post transfection with different mRNA Luc molecules. One way ANOVA, n=8 in four independent experiments.

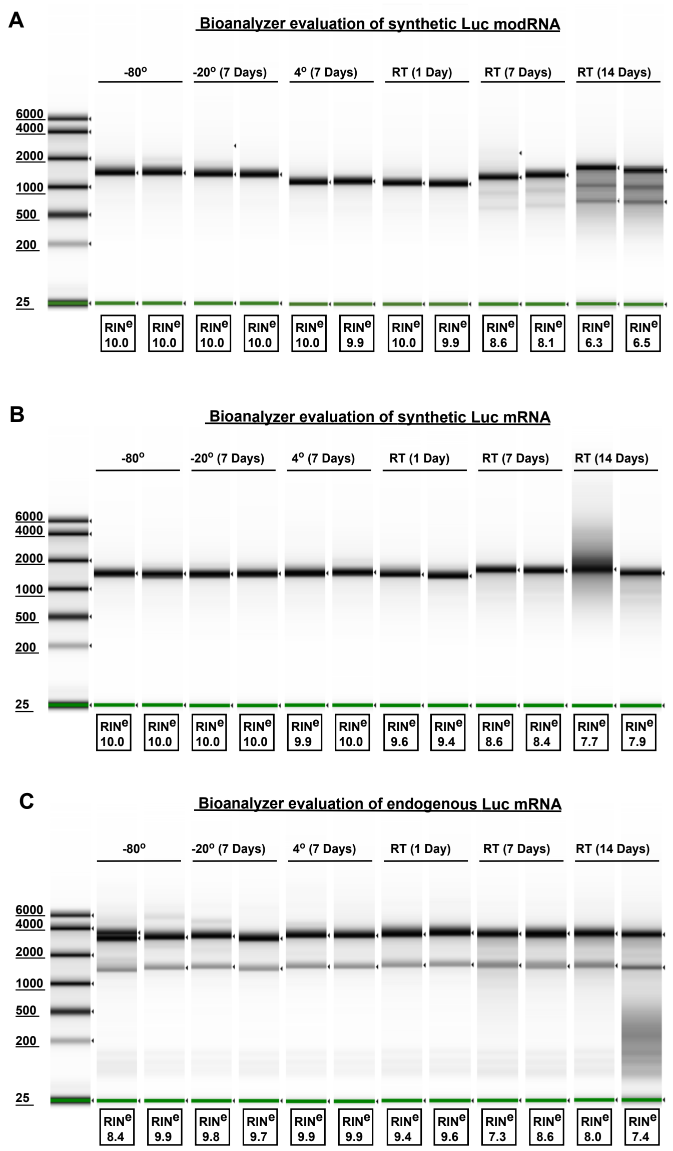

Figure S2: Evaluation of the integrity of Luc RNA molecules. Representative images of on-chip gel electrophoresis of synthetic Luc modRNA (**A**), synthetic Luc mRNA (**B**) and endogenous Luc mRNA (**C**) after storage in various conditions. Analysis was performed using bioanalyzer.

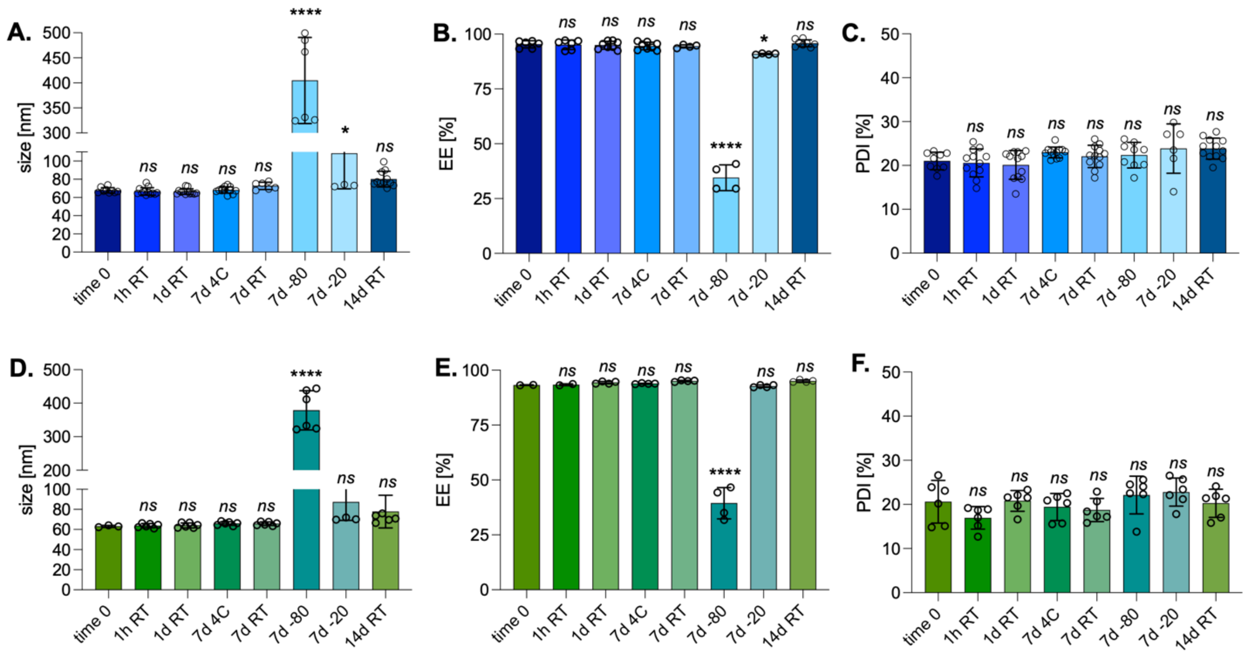

G.

**Luc**								
**size [nm]**	**time 0**	**1h RT**	**1d RT**	**7d 4C**	**7d RT**	**7d -80C**	**7d -20C**	**14d RT**
Mean	67.9	66.7	66.4	68.2	72.7	404.8	108.4	80.2
SD	3.0	4.1	3.2	3.5	4.0	85.8	39.0	8.7
**EE [%]**								
Mean	95.4	95.2	94.9	94.6	94.4	34.5	90.9	95.8
SD	1.6	2.2	1.8	1.7	0.7	5.9	0.3	1.6
**PDI [%]**								
Mean	21.0	20.6	20.1	23.0	22.1	22.3	23.9	23.8
SD	2.0	3.2	3.3	1.2	2.5	2.9	5.6	2.4
**nGFP**								
**size [nm]**	**time 0**	**1h RT**	**1d RT**	**7d 4C**	**7d RT**	**7d -80C**	**7d -20C**	**14d RT**
Mean	63.1	63.8	64.1	65.9	65.7	378.8	87.3	77.8
SD	1.0	2.1	2.4	1.9	1.8	58.7	18.3	16.2
**EE [%]**	93.2	93.4	94.4	93.9	95.1	39.4	92.7	95.0
Mean	0.1	0.4	0.5	0.2	0.3	7.1	0.6	0.5
SD								
**PDI [%]**								
Mean	20.6	17.0	20.8	19.4	18.7	22.1	22.8	20.3
SD	4.8	2.6	2.4	3.1	2.6	4.3	3.2	3.2

Figure S3: Evaluation of the integrity of synthetic modRNA-LNP formulations in various storage conditions. Quality control of LNP-modRNA formulations containing either Luc (upper panel) or nGFP (lower panel) modRNA show size (A, D), encapsulation efficiency EE (B, E) and

polydispersity index PDI (C, F) after storage in various conditions. One way ANOVA, n= 4-8 in three (Luc) and two (nGFP) independent experiments. **G.** A table summarizing numeric values of quality control measurements (SD – standard deviation).
